# Investigating the causal effect of potential therapeutic agents for colorectal cancer prevention: a Mendelian randomization analysis

**DOI:** 10.1158/1055-9965.EPI-25-0875

**Published:** 2026-01-09

**Authors:** Ella Fryer, D Timothy Bishop, Peter T Campbell, Andrew T Chan, Loic Le Marchand, Christopher I Li, Victor Moreno, Marc J Gunter, Amanda I Phipps, Robert C Grant, Stephanie L Schmit, Richard M Martin, James Yarmolinsky, Philip Haycock

**Affiliations:** 1 MRC Integrative Epidemiology Unit, University of Bristol, Bristol, England, BS8 2BN, UK; 2 Population Health Sciences, Bristol Medical School, University of Bristol, Bristol, England, BS8 2BN, UK; 3 Leeds Institute of Cancer and Pathology, University of Leeds, Leeds, UK; 4 Department of Epidemiology and Population Health, Albert Einstein College of Medicine, Bronx, NY, USA; 5 Division of Gastroenterology, Massachusetts General Hospital and Harvard Medical School, Boston, Massachusetts, USA; 6 Channing Division of Network Medicine, Brigham and Women’s Hospital and Harvard Medical School, Boston, Massachusetts, USA; 7 Clinical and Translational Epidemiology Unit, Massachusetts General Hospital and Harvard Medical School, Boston, Massachusetts, USA; 8 Broad Institute of Harvard and MIT, Cambridge, Massachusetts, USA; 9 University of Hawaii Cancer Center, Honolulu, Hawaii, USA; 10 Public Health Sciences Division, Fred Hutchinson Cancer Center, Seattle, Washington, USA; 11 Unit of Biomarkers and Suceptibility (UBS), Oncology Data Analytics Program (ODAP), Catalan Institute of Oncology (ICO), L’Hospitalet del Llobregat, 08908 Barcelona, Spain; 12 ONCOBELL Program, Bellvitge Biomedical Research Institute (IDIBELL), L’Hospitalet de Llobregat, 08908 Barcelona, Spain; 13 Consortium for Biomedical Research in Epidemiology and Public Health ( CIBERESP), 28029 Madrid, Spain; 14 Department of Clinical Sciences, Faculty of Medicine and health Sciences and Universitat de Barcelona Institute of Complex Systems (UBICS), University of Barcelona (UB), L’Hospitalet de Llobregat, 08908 Barcelona, Spain; 15 Department of Epidemiology and Biostatistics, School of Public Health, Imperial College London, London, England, W2 1PG, UK; 16 Department of Epidemiology, University of Washington, Seattle, Washington, USA; 17 Division of Medical Oncology and Hematology, Princess Margaret Cancer Centre, University Health Network, Toronto, Canada; 18 Genomic Medicine Institute, Cleveland Clinic, Cleveland, OH, USA; 19 Department of Molecular Medicine, Cleveland Clinic Lerner College of Medicine of Case Western Reserve University School of Medicine, Cleveland, OH, USA; 20 NIHR Bristol Biomedical Research Centre, University Hospitals Bristol and Weston NHS Foundation Trust, University of Bristol, Bristol, England, BS8 2BN, UK

## Abstract

**Background:**

Conventional observational studies have identified several potential therapeutic agents that may lower risk of colorectal cancer development. However, these studies are susceptible to unmeasured and residual confounding and reverse causation, undermining robust causal inference.

**Methods:**

We used Mendelian randomization (MR), a genetic epidemiological method that can strengthen causal inference, to evaluate the effect of previously reported therapeutic agents on colorectal cancer risk, including medications, dietary micronutrients, and exogenous hormones. Genetic instruments were constructed using genome-wide association studies (GWASs) of molecular traits (e.g. circulating levels of protein drug targets, blood-based biomarkers of micronutrients, circulating levels of endogenous hormones). Using summary statistics from these GWASs and a colorectal cancer risk GWAS (cases=78,473, controls=107,143), we employed Wald ratios and inverse-variance weighted models to estimate causal effects.

**Results:**

We found evidence for associations of genetically-proxied elevated omega-3 fatty acids (OR 1.10; 95% CI 1.03, 1.18; *p*=6.20x10^-3^) and reduced plasma ACE levels (OR 1.08; 95% CI 1.03, 1.13; *p*=9.36x10^-4^) with colorectal cancer risk. Findings for ACE inhibition were consistent across sensitivity analyses.

**Conclusions:**

Reduced plasma ACE levels were robustly linked to increased colorectal cancer risk. Further work is required to better understand the mechanism behind this finding and whether this translates to adverse effects via medication use (i.e. ACE inhibitors).

**Impact:**

These findings provide updated evidence on the role of previously reported therapeutic agents in colorectal cancer risk, helping to prioritise further evaluation of those agents with potential aetiological roles in cancer development.

## Introduction

Colorectal cancer presents an increasing public health burden, with over 1.9 million new cases globally in 2022, predicted to rise to 3.2 million annually by 2040 (1, 2). Incidence has been increasing in low-and middle-income countries in particular, correlated with economic growth and adoption of a western lifestyle, and amongst a younger demographic (i.e. under the age of 50) in high-income countries (3–6). An individual’s risk of developing colorectal cancer is influenced by several factors, including hereditary genetic mutations and family history, which account for 5-10% of colorectal cancer cases, and a number of environmental and lifestyle factors (7–9). Whilst many of these risk factors are modifiable, such as elevated body mass index (BMI), physical inactivity, and alcohol consumption; lifestyle and behavioural changes can be difficult to implement. Screening and removal of polyps during colonoscopy in high-income countries has been an effective strategy for reducing colorectal cancer incidence and mortality in average-risk groups; however, given incomplete uptake (10–12), screening alone is insufficient to tackle overall disease burden, and additional prevention strategies are needed (13).

Therapeutic prevention is one strategy that could be used to reduce an individual’s risk of developing colorectal cancer. Therapeutic prevention involves administering a synthetic, natural, or biological agent that can prevent, reverse or delay the onset of disease (14, 15). These agents could be used for primary prevention in a healthy population (i.e. showing no signs of disease) to reduce their overall risk, or in individuals who have had polyps (a precursor to colorectal cancer) removed during colonoscopy screening, known as secondary prevention (15, 16). Examples of successful preventive therapy agents for colorectal cancer include aspirin, for which a daily dose has been shown to reduce cancer incidence (17). Given the potential adverse effects of aspirin, including gastrointestinal bleeding, aspirin is currently only recommended for groups at high-risk of developing colorectal cancer (e.g. individuals with Lynch syndrome), and so effective therapeutic agents with more favourable safety profiles for cancer prevention are needed (18). There has been great success of preventive therapy in other disease areas including cardiovascular disease; however, there are fewer examples of successful preventive therapy for cancer, in part due to the longer timeframe of cancer development and limited availability of short-term biomarkers of drug target efficacy (14, 15). For clinical trials to be conducted, significant epidemiological evidence is required to select preventive agents for testing (15). There have been many preventive agents linked to reduced risk of colorectal cancer in the observational epidemiological literature that are pharmacologically actionable (i.e. given as a supplement or drug), including dietary micronutrients, medications and exogenous hormones. However, findings from conventional observational studies can be susceptible to biases such as confounding, due to either unmeasured or imprecisely measured confounders, and reverse causation, where associations are driven by the outcome influencing the presumed exposure. It can therefore be difficult to distinguish true causal effects from spurious correlations, and the suitability of these agents as intervention targets is unclear.

Mendelian randomization uses germline genetic variants to instrument exposures of interest, here dietary micronutrients, medications and hormones. As germline genetic variants are randomly allocated at meiosis and fixed from conception, conventional sources of bias such as confounding and reverse causation should be minimised, strengthening causal inference (19–21). We used Mendelian randomization to reassess these previously identified observational relationships and provide evidence to support their causal nature (19–21).

## Materials and Methods

**Figure 1:**
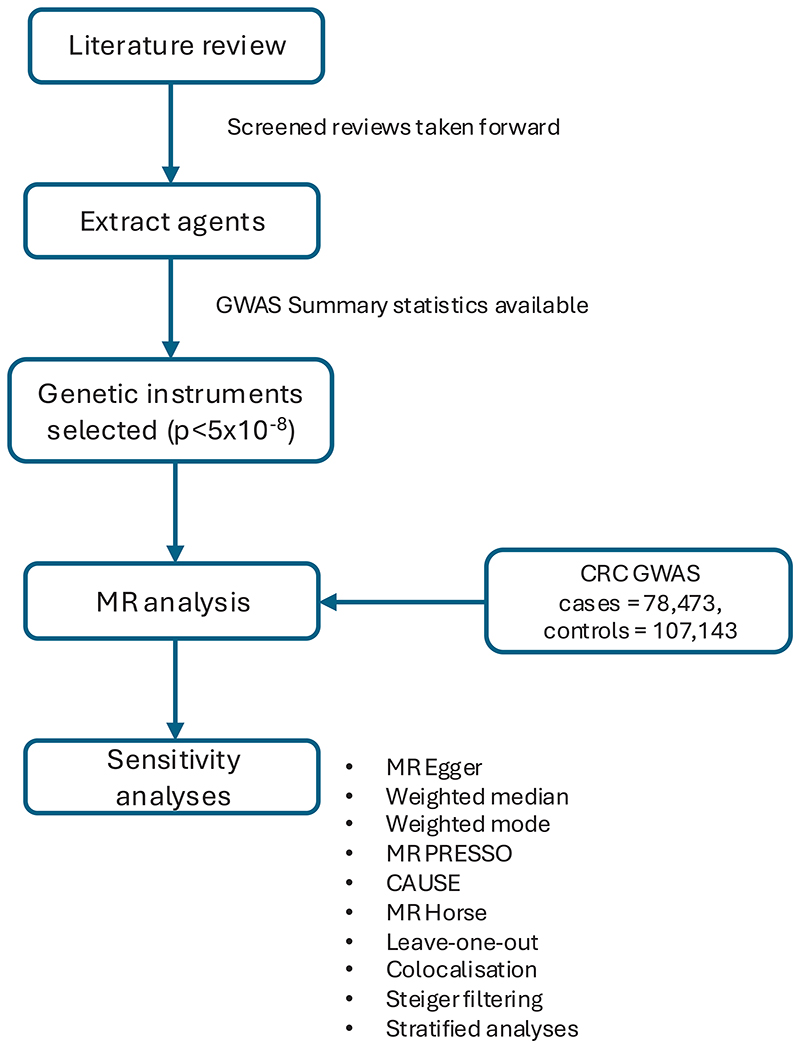
Outline of methods used in these analyses Overview of methods and sensitivity analyses conducted in this study. Abbreviations: GWAS = genome-wide association study; MR = Mendelian randomization; colorectal cancer = colorectal cancer; FDR = false discovery rate; PRESSO = Pleiotropy RESidual Sum and Outlier; CAUSE = Causal Analysis Using Summary Effect estimates; Horse after the horseshoe prior.

We used a two-sample MR framework to evaluate the causal relevance of preventive agents that have been reported to be associated with reduced colorectal cancer risk in observational studies. We have previously published a protocol detailing our methods and proposed analyses for this study (Figure 1) (22). In brief, we first conducted a literature search of reviews of therapeutic prevention and colorectal cancer risk to identify potential preventive agents. The search strategy is available in our protocol (22) and articles were included if they reviewed observational studies conducted in humans. All reported preventive agents were extracted from each review. For each agent that we identified, we attempted to generate a genetic instrument for a corresponding molecular trait (e.g. for drugs, this was circulating levels of protein drug targets; for dietary micronutrients, this was blood-based biomarkers of micronutrients; and for exogenous hormones, this was circulating levels of endogenous hormones). For instrument selection, we searched for genome-wide association studies (GWASs) using the GWAS catalogue (23), the IEU Open GWAS (24), PubMed and the preprint servers medRxiv/bioRxiv, to identify the largest GWAS conducted in individuals of European ancestry and with complete summary data available. Study specific covariates and reported units for each study are available in Supplementary Table 1. For any exposures not reported in standardised units, we transformed these prior to analyses. We then conducted a two-sample MR analysis of these potential preventive agents and colorectal cancer risk, using a GWAS meta-analysis of colorectal cancer risk available in individuals of European ancestry (cases=78,473, controls=107,143) (adjusted for age, sex and 10 genetic principal components of ancestry (PCs)) (25). Between study heterogeneity was calculated using the *I*^*2*^ statistic and variants with *I*^*2*^ >65% were excluded. Effect estimates for genetically proxied micronutrients and hormones were scaled to reflect increasing levels, whilst for the protein drug targets, estimates were scaled to reflect decreasing levels, to mimic the hypothetical intervention (i.e. micronutrient supplementation, hormone replacement therapy, or inhibition of proteins by drugs). For all molecular traits found to have evidence for an association with colorectal cancer risk (defined as p<0.05), and for which we had >10 single-nucleotide polymorphisms (SNPs) in the instrument, we conducted ‘pleiotropy-robust’ methods to examine sensitivity of results to horizontal pleiotropy bias (i.e. when a genetic variant influences the outcome, either directly or indirectly, independently of the exposure). For any protein drug targets found to have an effect on colorectal cancer risk, we performed genetic colocalisation analyses using the ‘coloc’ package in R (26), to determine if there is a shared causal variant, which is necessary, although not sufficient, to infer a causal relationship between these traits. The posterior probabilities for a number of configurations were calculated: H0 = neither the protein drug target or colorectal cancer has a genetic association in the region, H1 = only the protein drug target has a genetic association in the region, H2 = only colorectal cancer has a genetic association in the region, H3 = both the protein drug target and colorectal cancer are associated, but with different causal variants, H4 = both the protein drug target and colorectal cancer are associated and share a single causal variant. A posterior probability of ≥0.5 was used to indicate support for a configuration.

We detail here where we have deviated from, or made additions to, our published protocol. Firstly, our protocol specified that to instrument protein drug targets and biomarkers we would allow weakly correlated SNPs (r^2^<0.1) in our genetic instrument and account for this by using a generalised IVW method (27), using a correlation matrix generated from a reference panel of 10,000 individuals from UK Biobank. To add to this, when instrumenting drug target biomarkers, we used a ±250kb region around the cognate gene for the protein drug target to generate these instruments. We deviated from this approach when constructing instruments for ACE inhibition, where we instrumented plasma levels of ACE using a region ±1MB either side of the *ACE* gene (28) and an LD (linkage disequilibrium) r^2^<0.001 to select independent SNPs. This approach is consistent with previously published work, investigating the effect of genetically proxied ACE inhibition using serum ACE levels, on cancer risk (29). For all other exposures (e.g. micronutrients and hormones), we used a genome-wide significance (p<5x10^-8^) threshold and an r^2^<0.001 to select instruments, as stated in the protocol. Secondly, to account for potential sex-specific genetic influences on hormone levels, we conducted MR analyses of hormone levels using colorectal cancer data stratified by sex (female: cases=26,843, controls=32,820; male: cases=31,288, controls=34,527) (30), which was not previously stated in the protocol. Thirdly, our protocol specified that a false-discovery rate (FDR) correction of 5% was used to define strong evidence. Given that our hypotheses were guided by observational evidence, we deviated from the protocol and interpreted a p-value threshold of <0.05 as indicative of some evidence of an association, carrying forward all findings reaching this threshold for sensitivity analyses. The FDR corrected finding, along with the results of sensitivity analyses, were used to inform our interpretation of findings in the discussion. We also conducted additional sensitivity analyses for correlated horizontal pleiotropy that was not previously specified in the protocol, including MR-Horse (31) and CAUSE (32). Correlated pleiotropy occurs when the genetic variants used to instrument an exposure, affect a confounder that acts on both the exposure and outcome. CAUSE attempts to determine if genetic associations for two traits are consistent with a causal effect by comparing whether a sharing model, allowing for horizontal pleiotropic effects, or a causal model fit the data better (32). Regarding the colocalisation analyses, we used the default parameters (e.g. the prior probabilities of the SNP being associated with the exposure, the outcome or both traits is specified as 1x10^-4^, 1x10^-4^ and 1x10^-5^, respectively), as specified in the protocol. We did not specify in the protocol that ±100kb either side of the lead SNP was used to define the region and a more stringent threshold of 5x10^-6^ was used to test p12 (the SNP being associated with both traits), as a sensitivity analysis. For any findings with evidence of an effect on colorectal cancer risk in the main analysis, we conducted MR analyses using colorectal cancer data stratified by anatomical subsite, including colon (cases=32,002, controls=64,159), rectal (cases=16,212, controls=64,159), proximal (cases=15,706, controls=64,159), and distal (cases=14,376, controls=64,159) (30), as specified in the protocol. Additionally, we conducted analyses restricted to early age at onset (<50 years at diagnosis) (cases=6,176, controls=65,829) (33). In addition, a z-test was used to assess differences in findings between anatomical subsites (i.e. colon and rectal, distal and proximal) and age at onset (i.e. early onset and overall), as well as differences between sexes in the hormone analysis. When performing z-tests we accounted for potential sample overlap in control groups by calculating decoupled standard errors for effect estimates (34–36). There was overlap between cases of early onset and overall colorectal cancer, however we did not account for this in the z-test.

The Strengthening the Reporting of Observational Studies in Epidemiology using Mendelian Randomization (STROBE-MR) (37) guidelines were used to structure the reporting of this study.

## Results

### Literature review

We identified over 40 potential preventive agents in our literature review (Supplementary Table 2). Of these, 18 had a corresponding molecular trait (e.g. levels of protein drug targets, circulating dietary biomarkers and circulating levels of endogenous hormones) that could be instrumented and for which GWAS summary statistics of direct measures of these traits were available in individuals of European ancestry (Table 1). Characteristics of genetic variants used to instrument each trait and estimates of r^2^ and F-statistic are presented in Supplementary Table 3.

### Two-sample MR analyses

**Figure 2:**
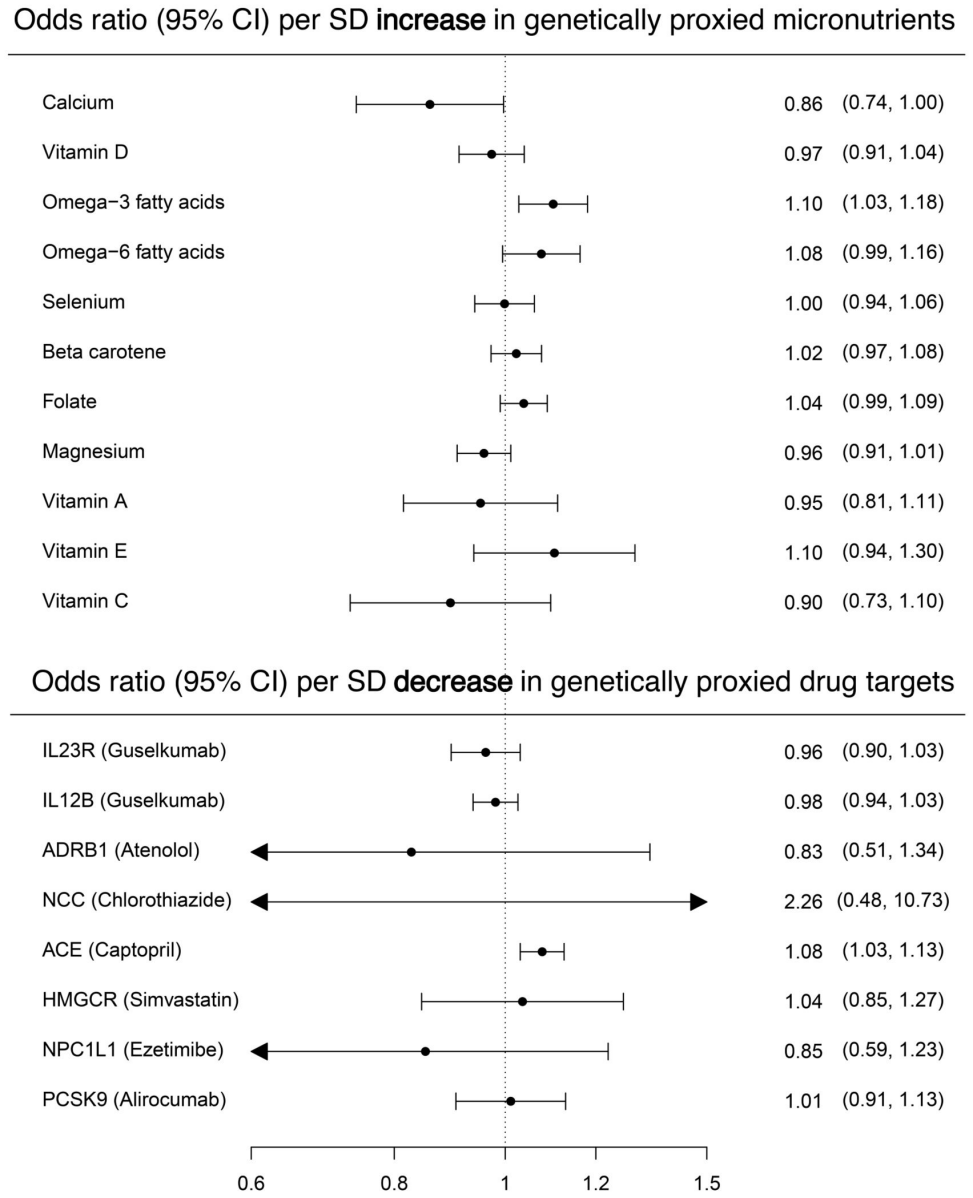
Effect estimates from initial Mendelian randomization analyses of circulating micronutrients and protein drug targets on colorectal cancer risk. Effect estimates are presented as odds ratios (ORs) per standard deviation (SD) unit increase (for micronutrients) or decrease (for protein drug targets) in genetically proxied exposure (95% confidence intervals). The odds ratio was calculated using the multiplicative random effects inverse variance weighted (IVW) method, except where the number of SNPs was limited (e.g. ≤3) in which case the fixed effects IVW was used. For those exposures instrumented by one SNP, the Wald ratio was used. Abbreviations: CI = confidence interval; SD = standard deviation; IL23R = interleukin 23 receptor; IL12B = interleukin 12B; ADRB1 = β1 adrenoceptor; NCC = ACE = angiotensin I converting enzyme; HMGCR = 3-hydroxy-3-methylglutaryl-CoA reductase; NPC1L = NPC1 like intracellular cholesterol transporter 1; PCSK9 = proprotein convertase subtilisin/kexin type 9; MR = Mendelian randomization; colorectal cancer = colorectal cancer.

We took all circulating micronutrients and protein drug targets for which we could construct genetic instruments and, using MR, tested their effect on colorectal cancer risk (Figure 2) (Supplementary Table 4). Effect estimates are presented as follows: odds ratios (ORs) per standard deviation (SD) unit increase (for micronutrients and hormones) or decrease (for protein drug targets) in genetically proxied exposure; 95% confidence intervals; p-value.

**Figure 3:**
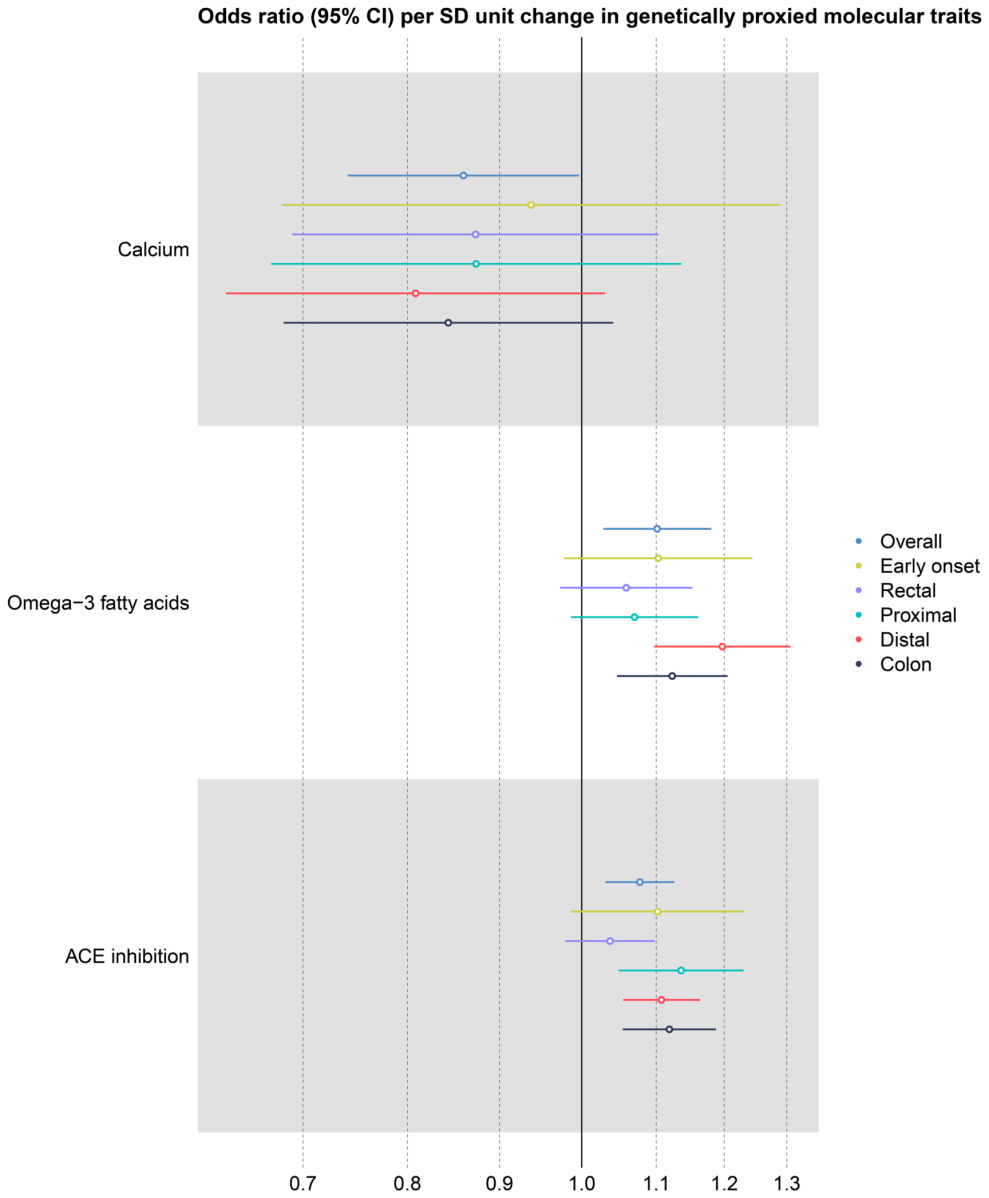
Mendelian randomization effect estimates stratified by anatomical subsite and restricted to early age at onset for micronutrients and drug targets found to have an effect on overall colorectal cancer. Effect estimates are presented as odds ratios (ORs) per standard deviation (SD) unit increase (for micronutrients) or decrease (for protein drug targets) in genetically proxied exposure (95% confidence intervals). The odds ratio was calculated using the multiplicative random effects inverse variance weighted (IVW) method, except where the number of SNPs was limited (e.g. ≤3) in which case the fixed effects IVW was used. For those exposures instrumented by one SNP, the Wald ratio was used. Effect estimates are scaled to reflect increasing micronutrient levels and decreasing protein levels. Abbreviations: CI = confidence interval; SD = standard deviation; ACE = angiotensin converting enzyme.

We initially found evidence that genetically proxied omega-3 fatty acids increased colorectal cancer risk (1.10; 1.03, 1.18; 6.20x10^-3^). This finding was generally consistent across ‘pleiotropy-robust’ sensitivity analyses (i.e. confidence intervals for all estimates overlapped) (Supplementary Table 5) but just exceeded the FDR corrected p-value threshold (Supplementary Table 4). Leave-one-out analyses indicated that a single SNP, rs174564, may be driving the effect estimate for omega-3 fatty acids on colorectal cancer risk, as removal of this SNP attenuated the effect to the null (1.02; 0.92, 1.13; 0.72), although confidence intervals did overlap with the overall effect estimate (Supplementary Table 6). We found strong evidence that a decrease in plasma ACE levels, the mechanism of action of ACE inhibitors, increased colorectal cancer risk (OR per unit decrease in inverse-rank normalized protein expression: 1.08; 1.03, 1.13; 9.36x10^-4^) (Figure 2). There was an insufficient number of independent SNPs in the genetic instrument to conduct pleiotropy-robust analyses on ACE inhibition, but this result met the FDR corrected p-value threshold. We found some evidence that the effect of ACE inhibition was stronger in colon cancer than rectum, (1.12, 1.05, 1.19; 2x10^-4^ and 1.04; 0.98, 1.10; 0.213, respectively) (pdiff=0.071)) (Figure 3, Supplementary Table 7-8). We found some evidence that circulating calcium levels had a protective effect on colorectal cancer risk (0.86; 0.74, 1.00; 0.045), which was consistent across anatomical subsites (Figure 3). Although this finding did not meet the FDR corrected p-value threshold, it was consistent across most of the pleiotropy-robust methods (Supplementary Table 5), except for CAUSE which did not find evidence for a causal relationship (Supplementary Table 9).

**Figure 4:**
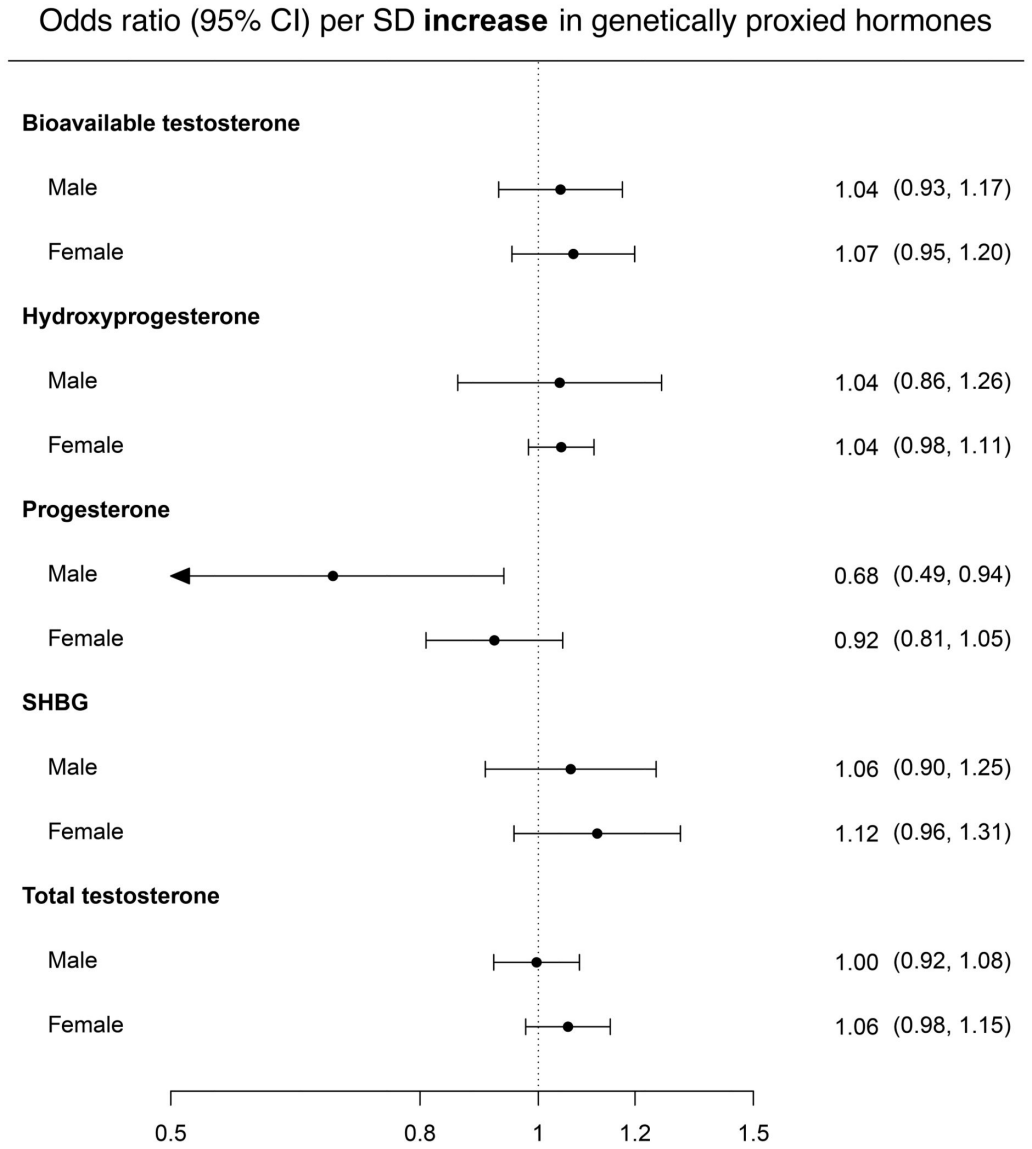
Effect estimates from Mendelian randomization analyses of circulating hormones on colorectal cancer risk in men and women. Effect estimates are presented as odds ratios (ORs) per standard deviation (SD) unit increase in genetically proxied hormone levels (95% confidence intervals). The odds ratio was calculated using the multiplicative random effects inverse variance weighted (IVW) method, except where the number of SNPs was limited (e.g. ≤3) in which case the fixed effects IVW was used. For those exposures instrumented by one SNP, the Wald ratio was used. Effect estimates are scaled to reflect increasing levels of circulating hormones. Abbreviations: SD = standard deviation; SHBG = sex hormone-binding globulin.

In analyses examining endogenous hormones we found evidence that genetically-proxied circulating progesterone had a protective effect on colorectal cancer risk in men (0.68; 0.49, 0.94; 0.019) (Figure 4) (Supplementary Table 10). However, this finding did not meet the FDR-corrected p-value threshold. We found little evidence of an effect of any other hormones on colorectal risk in either men or women. Progesterone in men was instrumented using 1 SNP, so we therefore could not conduct pleiotropy-robust sensitivity analyses.

### Multivariable Mendelian Randomization

The univariable IVW estimated effect for omega 3 fatty acids on colorectal cancer risk was OR=1.10, 95% CIs 1.03, 1.18, p=6.20x10^-3^. Given the genetic correlation between omega-3 and omega-6 fatty acids, estimated previously using LD score regression to be over 60% (38), and that the *FADS* SNP, rs174564, is strongly correlated with metabolism of both omega-3 and omega-6 fatty acids, it can be difficult to disentangle their independent effects on an outcome. We performed multivariable MR (MVMR) analyses to estimate the effect of omega-3 fatty acids whilst adjusting for omega-6 fatty acids (Supplementary Table 11). Given that the *FADS* SNP, rs174564, may have been driving the effect estimate for omega-3 fatty acids on colorectal cancer in the main analysis, we repeated this analysis removing this SNP from the omega-3 instrument. We also recalculated the univariable IVW estimate for omega-3 on colorectal cancer risk with this same SNP removed (Supplementary Table 11). The MVMR effect estimate for omega-3 with rs174564 included (OR=1.07, 95% CIs=0.96, 1.18, p=0.24) and excluded (OR=1.00, 95% CIs=0.86, 1.17, p=1.00) attenuated towards the null. The univariable IVW estimate for omega-3 excluding rs174564 (OR=1.04, 95% CIs=0.95, 1.14, p=0.19) also attenuated to the null.

### Steiger filtering

Steiger filtering can test the causal direction of SNP effects to evaluate if effect estimates are being driven by reverse causation (i.e. where the causal effect of a SNP on the exposure is mediated by the outcome). If the variance explained by a SNP was larger for the molecular trait than for colorectal cancer, this was consistent with a scenario where the molecular trait causally influences colorectal cancer risk, rather than the reverse. For ACE, omega-3 fatty acids and calcium, all SNPs were found to explain more of the variance in the molecular trait, consistent with the molecular trait causally influencing colorectal cancer.

### Colocalisation

**Figure 5:**
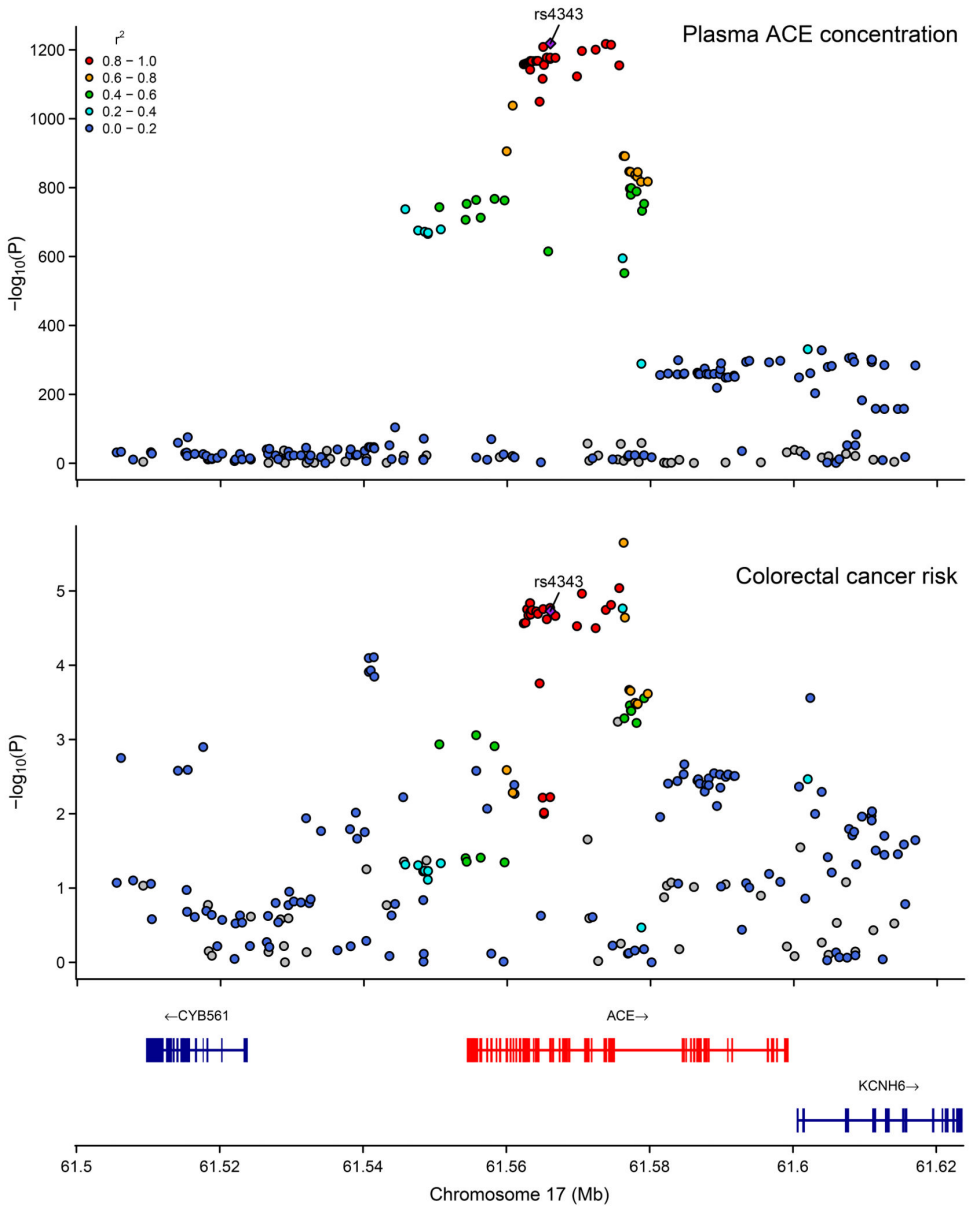
Regional association plots showing association of genetic variants with ACE inhibition and colorectal cancer risk ±100kb from the lead SNP (rs4343) used to instrument ACE. Regional association plots showing the genomic location of variants and their association (-log10(P)) with levels of ACE and colorectal cancer risk, generated using the LocusCompareR package. Posterior probability for a shared causal variant associated with both plasma ACE levels and colorectal cancer risk was 0.94, the regional association plots do not appear to support the presence of multiple independent variants driving the associations with either trait. Abbreviations: ACE = angiotensin-converting enzyme; chr = chromosome; Mb = megabase; colorectal cancer = colorectal cancer.

The colocalisation analyses of ACE inhibition and colorectal cancer risk found strong evidence that there is a shared causal variant in this region (posterior probability H4=0.94) (Figure 5) (Supplementary Table 12). This finding was consistent when using a more stringent threshold for the prior probability that the SNP was associated with both traits (e.g. p12=5x10^-6^) (posterior probability H4=0.89).

We attempted to perform SUSIE, which allows for multiple causal SNPs in the region, but no credible sets were found for colorectal cancer risk in the ACE locus as no SNPs in the colorectal cancer dataset reached the default minimum p-value threshold (1x10^-6^). SUSIE does not assess for evidence of an association in datasets where no SNPs fall below the minimum p-value threshold.

## Discussion

This Mendelian randomization analysis investigated the effects of genetically proxied circulating micronutrients, hormone levels, and inhibition of protein drug targets on colorectal cancer risk. To our knowledge, this is the largest and most comprehensive appraisal of previously reported therapeutic agents for colorectal cancer prevention, conducted in individuals of European ancestry using Mendelian randomization. We found strong evidence that genetically proxied inhibition of ACE increased colorectal cancer risk. We initially found evidence that genetically proxied elevated circulating omega-3 fatty acids increased colorectal cancer risk, however further analysis suggested other factors may have been driving this effect. We also found weak evidence for a protective effect of genetically proxied elevated circulating calcium levels.

We found strong evidence that genetically proxied ACE inhibition had an adverse effect on colorectal cancer risk, supported by genetic colocalisation of these traits around the *ACE* gene region. There was suggestive evidence that this effect was stronger in colon cancer, compared to rectal, however these analyses had limited statistical power. These findings are consistent with a previously published MR study investigating the effect of genetically proxied inhibition of antihypertensive drugs targets and a number of cancers, including colorectal cancer (29). The latter study differed in the method employed for instrument generation, permitting correlated SNPs (r^2^ <0.1) located within 100kb from the *ACE* gene (resulting in 14 SNPs) from a smaller GWAS of serum ACE levels (n=4,174) than used in the current study. The previous study also used summary data from a smaller GWAS for colorectal cancer risk (cases=58,221, controls=67,694) that made up a subset of the larger GWAS used in the current study (cases=78,473, controls=107,143), which enabled more precise effect estimation in the current study. Observational studies have found conflicting evidence on the association of ACE inhibitor use and colorectal cancer risk, for example; a large population-based case-control study (cases=15,560, controls=62,525) reported an increased risk of colorectal cancer (OR: 1.30, 95% CI: 1.22, 1.39) among long-term ACE inhibitor users (defined as 1000 daily doses within the past 5 years) as compared to age and gender matched controls (39). Whilst a large, retrospective, cohort study of 1,693,297 people (cases=28,460) found no evidence for an association of cumulative duration of ACE inhibitor use with colorectal cancer risk (HR: 1.03, 95% CI: 0.99, 1.07) (40). A dose response meta-analysis of 7 observational studies, including case-control and cohort studies (total cases=15,220, total controls=1,565,018) found an association of ACE inhibitor use with reduced colorectal cancer risk (RR: 0.81, 95% CI: 0.70, 0.92), however there was substantial heterogeneity between studies (*I*^2^=71.1%) (41). Given that cancer is not the indication for ACE inhibitors, there have not been any RCTs of ACE inhibitor use and colorectal cancer risk specifically. A recent unpublished study investigating the effects of several drugs and cancer risk using a target trial emulation framework, found that Captopril, the generic name of an ACE inhibitor, was associated with an increased colorectal cancer risk (HR = 2.15, 95% CI = [1.81, 2.57]), as compared to control drugs (medRxiv 2024.05.29.24308170). As this finding was generated using an alternative methodology and was consistent with the current study, it provides “triangulation” of the link between genetically proxied ACE inhibition and colorectal cancer risk.

The potential carcinogenic mechanisms of ACE inhibition are difficult to pinpoint given that ACE has a number of functions and effects in the body. ACE is primarily responsible for converting angiotensin I into angiotensin II, however it is capable of cleaving several other substrates (42). Bradykinin is one of these substrates and is involved in tissue injury and inflammation, as well as potentially influencing the tumour microenvironment, so increased levels, due to inhibition of ACE, may therefore play a role in tumorigenesis (43). Substance P is another substrate cleaved by ACE, which is involved in stimulating pro-inflammatory cytokine production and binds the NK-1 receptor, found on colon adenocarcinoma cells, to facilitate cell proliferation and migration of tumour cells (43). Angiotensin II can regulate levels of transferrin receptors which have been found to promote colon tumorigenesis and progression (44, 45), which could suggest a potential mechanism for increased risk of colon cancer over rectal. It would be valuable in future work to further investigate these potential mechanistic pathways in relation to ACE inhibition and increasing cancer risk.

Genetically-proxied higher levels of circulating omega-3 fatty acids were found to have an adverse effect on colorectal cancer risk which is inconsistent with previous meta-analyses of observational studies that have found an inverse association of higher omega-3 fatty acids on colorectal cancer risk (46–49). However, it can be difficult to disentangle the effects of omega-3 and omega-6 fatty acids in MR analyses given the large genetic overlap between the fatty acids. This was demonstrated by the MVMR analyses finding attenuated effect estimates for omega-3 fatty acids when adjusting for omega-6. In addition to this, in the leave-one-out analysis, a single SNP, rs174564, was identified as driving the MR effect estimate. After removing rs174564 from the analysis, little effect of omega-3 on colorectal cancer risk was found. This SNP is located in the fatty acid desaturase (*FADS*) gene region, suggesting that activity of this enzyme accounts for the effect on colorectal cancer risk. As the desaturase enzymes encoded by the *FADS* genes are involved in rate-limiting steps in the biosynthesis of both omega-3 and omega-6 fatty acids, it is not clear if the effect of *FADS* variants on colorectal cancer risk is independent of omega-6. Previous work has looked at individual omega-3 fatty acids and colorectal cancer risk and also found little evidence for an effect after removing SNPs from the *FADS* region, including for overall increased omega-3 fatty acids (OR: 1.11, 95% CIs: 0.97-1.28, p=0.13) (36). An RCT found little evidence for an effect of supplementation with omega-3 fatty acid, eicosapentaenoic acid (EPA), on risk of having any colorectal adenomas (RR: 0.98, 95% CIs: 0.87-1.12), although secondary analyses did identify an effect on reduction of number of colorectal adenomas (incidence rate ratio (IRR): 0.86, 95% CIs: 0.74-0.99) (50).

We found weak evidence that genetically proxied circulating calcium levels had a protective effect on colorectal cancer risk and that the direction of effect remained consistent across sensitivity analyses. The modest effect we observed in this study was consistent across anatomical subsites, so it appears that this effect was not driven by an effect at a particular site. There has been some evidence from several systematic reviews and meta-analyses of randomized controlled trials (RCTs) that calcium supplementation has a modest preventive effect on recurrent colorectal adenomas. One systematic review and meta-analysis looked at four randomised, double-blind, placebo-controlled trials of calcium supplementation (ranging from 1200-2000 mg/d) with the number of participants ranging from 194 to 1523 (total participants in calcium group=1487, total participants in placebo group=1497) (51). This study found an overall modest protective effect of calcium supplementation on recurrence of colorectal adenomas (random-effects risk ratio (RR) = 0.87, 95% CI: 0.77-0.98, I^2^=38.7%) (51). Another systematic review and meta-analysis included two trials comparing supplemental calcium (ranging from 1200-2000 mg/d) versus placebo for recurrence of adenomas, in 354-832 participants (total participants in calcium group=585, total participants in placebo group=601) (52). This analysis also found a risk reduction in developing an adenoma in the calcium supplementation arm compared to placebo (random-effects RR:0.82, 95%CI: 0.69–0.98, I^2^=0%) (52). Another systematic review of RCTs found contradictory results, with some studies finding increased risk or no effect, however, they were unable to perform a meta-analysis due to high heterogeneity between studies (53). A meta-analysis of cohort studies, including six studies with a total of 920,837 participants (including 8,839 colorectal cancer cases), also found a protective association of calcium supplementation with colorectal cancer risk (random effects RR per 300 mg/d = 0.91, 95% CI = 0.86-0.98, I^2^ = 67%) (54). A recent large prospective study of diet and colorectal cancer risk in 542,778 participants from the Million Women Study (12,251 incident cases over 16.6 years) found a strong inverse association for dietary calcium (RR per 300 mg/day = 0.83, 95% CI 0.77–0.89, p<1x10^-6^) (55). Serum calcium levels are tightly regulated and therefore may not be reflective of dietary intake (56, 57), which could explain why the magnitude of effect in the current study, where we used instruments generated from serum levels, was much smaller than studies measuring the effect of dietary or supplemental calcium. As previous prevention trials have focussed on recurrent adenomas and, given the latency period from colorectal adenoma to carcinoma (i.e. up to 10 years) (58, 59), large, longer-term primary prevention trials are required to confirm the potential protective effects of calcium supplementation on colorectal cancer development.

There are some considerations to acknowledge when conducting MR studies of nutritional factors. Firstly, sample sizes of GWASs are often small and so there are limited genetic variants available to instrument traits. For example, when constructing an instrument for beta-carotene, only one SNP was available. Subsequent MR studies may also be underpowered to detect effects given the small effect sizes of genetic variants on micronutrients, often due to a small heritable component of dietary micronutrient levels. There is often a lack of biological understanding of how genetic instruments influence micronutrient levels, limiting our ability to understand if variants are likely to be valid instruments, and subsequently our interpretation of MR findings. Circulating levels of biomarkers may also not reflect cellular levels of these biomarkers and often do not correlate with dietary intake (60), therefore limiting the application of findings to dietary interventions.

We found little evidence for an effect of genetically-proxied inhibition of the protein targets of lipid-lowering medication (e.g. statins), on colorectal cancer risk, and effect estimates were not consistent in direction. This is in disagreement with previous conventional observational studies that have found that lipid-lowering medications are associated with reduced colorectal cancer risk (61, 62). This relationship is also supported by a recent MVMR study that found an adverse effect of LDL cholesterol on colorectal cancer, adjusted for high-density lipoprotein (HDL) cholesterol and triglycerides (63). However, in the current analysis, we may have been underpowered to detect an effect of lipid-lowering drug targets on colorectal cancer given the far fewer number of genetic variants available to instrument these traits as compared to LDL cholesterol levels.

One strength of this analysis was that when proxying the effect of drug targets, genetic variants were selected that were located within genes that encode the drug’s protein target, which should minimise risk of horizontal pleiotropy bias and reverse causation. For most of the protein drug targets, with the exception of ACE, we instrumented a downstream biomarker of that particular drug (e.g. for lipid-lowering drugs this was reduced LDL cholesterol levels, and for antihypertensives this was reduced systolic blood pressure (SBP)). An advantage of using these variants instead of genetic variants associated with circulating levels of proteins (i.e. protein quantitative trait loci (pQTL)) is that these biomarkers are further along the causal pathway to the outcome, so these variants are more likely to mimic the drug effect. For ACE, we used variants associated with circulating plasma levels of this protein (pQTLs). This approach is consistent with previously published work, investigating the effect of genetically proxied ACE inhibition on cancer risk (29). The top variant (rs4343) in the ACE instrument explains a significant proportion of the variance in levels of this protein (9%) and is also associated with systolic blood pressure. A limitation of this finding is that the magnitude of the effect of ACE inhibition on colorectal cancer may be underestimated, since the effect of ACE inhibitors on lowering ACE levels is likely more potent than the genetic effect from the variants used here as proxies for ACE levels. Given that the genetic instrument used for ACE levels was associated with lower systolic blood pressure, people with these variants may be less likely to develop hypertension and subsequently less likely to be prescribed and treated with pharmacological ACE inhibitors.

There are lower rates of colorectal cancer in women as compared to men, and it has been suggested that sex hormones may play a role in this disparity (6, 64). However, we found little evidence for an effect of most of the hormones tested on colorectal cancer risk. We found evidence that progesterone has a protective effect on colorectal cancer risk in men, however the confidence intervals for this effect were wide, indicating uncertainty around the true estimate. This finding also does not explain the sex disparity observed in colorectal cancer rates. A RCT examining the effect of taking a daily combined oestrogen and progestin tablet on colorectal cancer risk in women initially found evidence for a protective effect on colorectal cancer risk (hazard ratio (HR): 0.63, 95% CIs: 0.43-0.92) (65). However, this study later found this finding was due to diagnostic delays and those in the treatment group were more likely to be diagnosed with advanced disease (66). This is in agreement with our findings, that showed little evidence for an effect of progesterone on colorectal cancer risk in women. This analysis was limited as we were unable to conduct further sensitivity analyses for these findings given the small number of SNPs available to instrument progesterone.

These analyses were restricted to individuals of European ancestry and therefore findings may not be generalisable to other ancestry groups. This is particularly relevant in the setting of therapeutic agents, given that they may have different pharmacokinetic/pharmacodynamic (PKPD) properties depending on germline pharmacogenomic variants that vary across populations (67, 68). In future, when GWAS data is available in different populations for the molecular traits examined here, there would be value in repeating these analyses using a more diverse cohort.

## Conclusion

These analyses found genetic evidence for a causal effect of several potential preventive agents on colorectal cancer risk. Reduced plasma ACE levels were robustly linked to increased colorectal cancer risk, and there was some evidence to support that elevated calcium and progesterone (in men) were inversely associated with colorectal cancer. However, given the limitations outlined here, further research is required to understand if our findings reflect the effects of the corresponding medication, supplement or exogenous hormone on colorectal cancer risk, and whether they can be translated to clinical interventions. Specifically, additional epidemiological evidence with non-overlapping sources of bias is needed to further evaluate the association between ACE inhibition and colorectal cancer risk, ideally with evidence from RCTs. Additionally, it would be useful to investigate whether different classes of ACE inhibitors have differing adverse outcomes in the long-term. This may be aided by further work investigating the potential mechanisms underlying the association found here. With new large-scale genetic and proteomic datasets becoming available, there is likely great potential for identifying novel targets for cancer prevention in future (69).

## Supplementary Material

STROBE-MR checklist

Supplementary Table 1

Supplementary Table 2

Supplementary Table 3

Supplementary Table 4

Supplementary Table 5

Supplementary Table 6

Supplementary Table 7

Supplementary Table 8

Supplementary Table 9

Supplementary Table 10

Supplementary Table 11

Supplementary Table 12

## Figures and Tables

**Table 1:** Potential preventive agents identified from reviews of observational studies of colorectal cancer risk and the corresponding molecular trait instrumented in Mendelian randomization analyses. Preventive agents identified in the literature that had a corresponding molecular trait that was deemed instrumentable. These agents included dietary micronutrients, drugs and endogenous hormones. For each drug, the biological target of the drug was identified as it’s molecular trait, (e.g. the levels of the protein that the drug targets). For dietary micronutrients and endogenous hormones, molecular traits were identified that correspond to the agent (e.g. blood-based biomarkers for vitamin levels). Abbreviations: GWAS = genome-wide association study; PMID = PubMed identifier; LDL = low-density lipoprotein; HMG-CoA = 3-hydroxy-3-methylglutaryl coenzyme A; NPC1L1 = NPC1 like intracellular cholesterol transporter 1; PCSK9 = proprotein convertase subtilisin/kexin type 9; IL23R = interleukin 23 receptor; IL12B = interleukin 12B; ACE = angiotensin-converting enzyme; SBP = systolic blood pressure; NCC = sodium chloride cotransporter; ADRB1 = β1 adrenoceptor; SHBG = sex hormone binding globulin.

Preventive agent	Molecular trait	GWAS PMID	Sample size
Dietary long-chain omega-3 polyunsaturated fatty acids	Circulating long-chain omega-3 polyunsaturated fatty acids	35692035 (70)	114999
Dietary long-chain omega-6 polyunsaturated fatty acids	Circulating long-chain omega-6 polyunsaturated fatty acids	35692035 (70)	114999
Dietary calcium	Serum calcium levels	33462484 (71)	313387
Dietary vitamin D	Serum 25 hydroxyvitamin D	32059762 (72)	443734
Dietary folate levels	Serum folate levels	30339177 (73)	2232
Dietary selenium	Circulating selenium levels	23720494 (74)	5477
Dietary vitamin A	Serum retinol levels	38374065 (75)	22274
Dietary vitamin C	Plasma vitamin C levels	33203707 (76)	52018
Dietary vitamin E	Circulating alpha-tocopherol levels	36635386 (77)	8192
Dietary β carotene	Circulating β carotene levels	19185284 (78)	1190
Dietary magnesium	Serum magnesium levels	20700443 (79)	15366
Statins	LDL cholesterol levels (due to HMGCR inhibition)	24097068 (80)	1320016
	LDL cholesterol levels (due to NPC1L1 inhibition)	24097068 (80)	1320016
	LDL cholesterol levels (due to PCSK9 inhibition)	24097068 (80)	1320016
Guselkumab	Plasma IL23 protein levels (i.e. IL23R inhibition, IL12B inhibition)	29875488 (81)	3301
Antihypertensives	Plasma ACE levels	37794186 (82)	54219
	SBP (due to NCC inhibition)	30224653 (83)	757601
	SBP (due to ADRB1 inhibition)	30224653 (83)	757601
Testosterone	Bioavailable testosterone levels	32042192 (84)	188507 (women) 178782 (men)
	Total testosterone levels	32042192 (84)	230454 (women) 194453 (men)
Sex hormone binding globulin	Serum SHBG levels	32042192 (84)	189473 (women) 180726 (men)
Progesterone/17-hydroxyprogesterone	Circulating progesterone levels	34822396 (85)	1877 (women) 2220 (men)
	17-hydroxyprogesterone levels	34822396 (85)	1329 (women) 2220 (men)

## Data Availability

We obtained publicly available summary genetic association data for colorectal cancer from GECCO (https://www.ebi.ac.uk/gwas/publications/36539618). Approval was received to use restricted summary genetic association data (including for sex, anatomical subsite and early age at onset specific colorectal cancer) from GECCO consortia after submitting a proposal to GECCO (kafdem@fredhutch.org). We obtained publicly available summary genetic association data for all exposures, the GWAS PMID for each study used is presented in Table 1 and details for accessing summary data can be found in each paper.
